# Momentum-space spin texture induced by strain gradient in nominally centrosymmetric SrIrO_3_ films

**DOI:** 10.1093/nsr/nwad296

**Published:** 2023-11-21

**Authors:** Minghui Gu, Haohao Sheng, Xiaofeng Wu, Mei Wu, Xiaoran Liu, Fang Yang, Zhongshan Zhang, Peng Gao, Zhijun Wang, Meng Meng, Jiandong Guo

**Affiliations:** Beijing National Laboratory for Condensed Matter Physics and Institute of Physics, Chinese Academy of Sciences, Beijing 100190, China; School of Physical Sciences, University of Chinese Academy of Sciences, Beijing 100190, China; Beijing National Laboratory for Condensed Matter Physics and Institute of Physics, Chinese Academy of Sciences, Beijing 100190, China; School of Physical Sciences, University of Chinese Academy of Sciences, Beijing 100190, China; Beijing National Laboratory for Condensed Matter Physics and Institute of Physics, Chinese Academy of Sciences, Beijing 100190, China; School of Physical Sciences, University of Chinese Academy of Sciences, Beijing 100190, China; International Center for Quantum Materials, and Electron Microscopy Laboratory, School of Physics, Peking University, Beijing 100091, China; Beijing National Laboratory for Condensed Matter Physics and Institute of Physics, Chinese Academy of Sciences, Beijing 100190, China; Beijing National Laboratory for Condensed Matter Physics and Institute of Physics, Chinese Academy of Sciences, Beijing 100190, China; Beijing National Laboratory for Condensed Matter Physics and Institute of Physics, Chinese Academy of Sciences, Beijing 100190, China; International Center for Quantum Materials, and Electron Microscopy Laboratory, School of Physics, Peking University, Beijing 100091, China; Beijing National Laboratory for Condensed Matter Physics and Institute of Physics, Chinese Academy of Sciences, Beijing 100190, China; School of Physical Sciences, University of Chinese Academy of Sciences, Beijing 100190, China; Beijing National Laboratory for Condensed Matter Physics and Institute of Physics, Chinese Academy of Sciences, Beijing 100190, China; Beijing National Laboratory for Condensed Matter Physics and Institute of Physics, Chinese Academy of Sciences, Beijing 100190, China; School of Physical Sciences, University of Chinese Academy of Sciences, Beijing 100190, China

**Keywords:** spin texture, spin–orbit coupling, transition metal oxides, strain gradient

## Abstract

Spin texture in **k**-space is a consequence of spin splitting due to strong spin–orbit coupling and inversion symmetry breaking. It underlies fertile spin transport phenomena and is of crucial importance for spintronics. Here, we observe the spin texture in **k**-space of nominally centrosymmetric SrIrO_3_ grown on NdGaO_3_ (110) substrates, using non-linear magnetotransport measurements. We demonstrate that the spin texture is not only induced by the interface, which inherently breaks the inversion symmetry in strong spin–orbit coupled SrIrO_3_ films, but also originates from the film bulk. Structural analysis reveals that thicker SrIrO_3_ films exhibit a strain gradient, which could be considered as a continuous change in the lattice constant across different layers and breaks the inversion symmetry throughout the entire SrIrO_3_ films, giving rise to the spin texture in **k**-space. First-principles calculations reveal that the strain gradient creates large spin-splitting bands, inducing the spin texture with anisotropy, which is consistent with our experimental observations. Our results offer an efficient method for inducing the spin textures in **k**-space.

## Teaser

Induce spin textures in k-space of a nominally centrosymmetric material by a new kind of inversion symmetry broken mechanism, and detect the spin textures by nonlinear magnetotransport measurements.

## INTRODUCTION

Spin textures in **k**-space underpin various spin transport phenomena, such as the spin-Hall effect (SHE) [1,2] and spin-charge conversion [3–7]. Understanding and manipulating spin textures is one of the central issues in the current spintronics research. Spin textures are the consequence of spin splitting due to strong spin–orbit coupling (SOC) and inversion symmetry breaking. Hexagonally warped helical spin textures can be induced by spin-momentum locked surface states in topological insulators [8–10]. Chiral spin textures can be also generated by spin-splitting energy bands and spin-polarized Fermi surfaces in Rashba systems [11–13]. Recently, a non-linear transport measurement has been used to detect spin textures in various materials such as topological insulator Bi_2_Se_3_, Dirac semimetal α-Sn, semiconductor Ge (111) and 2D electron gases (2DEGs) [14–20].

The induction of spin textures in centrosymmetric materials offers a significant expansion of applicable material systems for spintronics [21]. The representative way is to introduce interfaces, which can create structural inversion asymmetry in centrosymmetric materials, resulting in Rashba-split states [22] because of electrostatic potential gradients [23,24]. However, due to the low Rashba splitting energy, such as 3 meV (∼35 *k*_B_) of typical oxide 2DEGs LaAlO_3_/SrTiO_3_ [20], spin textures can only be detected at very low temperatures [17].

Given the strong SOC, 5*d*-transition metal oxides (TMOs) have been demonstrated to show the SHE with a large spin-Hall angle *θ*_SHE_. As a prototypical 5*d*-TMO, SrIrO_3_ (SIO) crystallizes in the orthorhombic perovskite structure with the space group *Pbnm* if it is synthesized under high pressure [25] or is in the epitaxial thin film form [26]. The *θ*_SHE_ of SIO films is reported to be in the range of 0.3–1.0 (with unit of *ћ*/2*e*) [4], which is much larger than the *θ*_SHE_ of conventional heavy metals such as Pt/Co, at ∼0.11 [27]. Due to the strong SOC and inherent inversion symmetry broken at the interface/surface of SIO films, a recent study suggests that the spin texture could be induced in nominally centrosymmetric SIO films [28]. The spin texture has been detected at room temperature, but its modulation still cannot be achieved since lattice strain was reported to not affect the underlying spin texture.

In this work, we report on the induced spin textures in **k**-space of nominally centrosymmetric SIO films, as well as the tuning of non-linear magnetoresistance (NLMR) signals. We identify the interface-induced spin texture in a fully strained SIO film by detecting the magnetic-field angular-dependent NLMR, which can be well understood by using theoretical calculation. However, for the thicker SIO film, the peak position of the magnetic-field angular-dependent second-order resistance shifts at varying temperatures, which is not observed in the fully strained SIO film. Through systematic structural analysis, we unveil that this anomaly in NLMR originates from the tuned spin texture induced by the strain gradient in the thicker SIO film. First-principles calculations indicate that the strain gradient leads spin-split bands and spin texture with anisotropy along $\overline {{{\bf \Gamma X}}} $ and $\overline {{{\bf \Gamma }}{{{\bf X}}}^{{\bf ^{\prime}}}} $, which is different from the interface-induced one. Our findings offer an efficient approach to inducing the spin texture in nominally centrosymmetric materials and pave the way for tuning the NLMR in spintronics devices.

## RESULTS AND DISCUSSION

**Figure 1.**
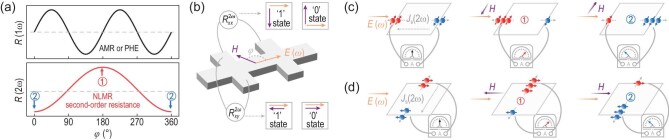
Schematic diagrams of non-linear magnetoresistance (NLMR). (a) Schematic curves of magnetic-field angular-dependent first-order resistance and second-order resistance. (b) NLMR shows a 2π periodic angular dependence, i.e. there exists a high-resistance state and a low-resistance state [① and ② in (a)], which could be considered as two logical states ‘0’ and ‘1’. Schematics of NLMR along both the (c) longitudinal ($R_{xx}^{2{\mathrm{\omega }}}$) and (d) transverse ($R_{xy}^{2{\mathrm{\omega }}}$) directions. When an electric field *E* (ω) is applied along a certain direction, a net spin current [*J*_s_(*E*^2^)] could be generated at the second order of the electric field [i.e. *J*_s_(*E*^2^), proportional to 2ω, left panel] due to spin-momentum locking (left panel). When an external magnetic field *H* is applied, the non-linear spin current is partially converted into a charge current *J*_e_(*E*^2^). A low-resistance [middle panel, corresponds to ② in (a)] or high-resistance [right panel, corresponds to ① in (a)] state would be generated if the magnetic field is antiparallel or parallel to *E* (ω).

Recently developed non-linear magnetotransport measurement has made it possible to map spin textures without using spin-/angle-resolved photoemission spectroscopy [14–20]. Phenomenologically, the dependence of the first-order resistance on the relative orientation between the magnetic field and the alternating current with frequency *ω* (generally called anisotropic magnetoresistance, AMR), which stems from crystalline anisotropy or magnetic anisotropy, exhibits a period of π or less (Fig. 1a) [29–31], while the second-order resistance could exhibit a 2π periodic angular dependence (Fig. 1a), i.e. non-reciprocal and rectification characteristics (Fig. 1b), which depends on the zero-field spin texture as well as the relative direction between the magnetic field and the electric field [15]. In strong SOC systems with spin-momentum locking, the non-linear response originates from a second-order spin current ${{\bf J}}_{{\bf S}}^{2\omega }$ (${{\bf J}}_{{\bf S}}^{2\omega } \propto {{{\bf E}}}^2$) induced by the second-order correction to the electron distribution in the applied electric field **E** [32], which could be simultaneously generated along either the longitudinal or the transverse direction (left panels of Fig. 1c and d). Under further breaking time inversion symmetry via applying the external magnetic field **H**, the second-order correction to the electron distribution is shifted in **k**-space and two electron fluxes with opposite spin directions cannot be compensated, causing ${{\bf J}}_{{\bf S}}^{2\omega }$ to be partially converted into a second-order charge current ${{\bf J}}_{{\bf e}}^{2\omega }$ (middle and right panels of Fig. 1c and d). Thus, by extracting the second-order voltage ($V_{xx}^{2\omega }$ or $V_{xy}^{2\omega }$), high or low second-order (longitudinal or transverse) resistance states ($R_{xx}^{2\omega } = V_{xx}^{2\omega }/{I}^\omega $ or $R_{xy}^{2\omega } = V_{xy}^{2\omega }/{I}^\omega $) would be detected with opposite **H** (Fig. 1b).

**Figure 2.**
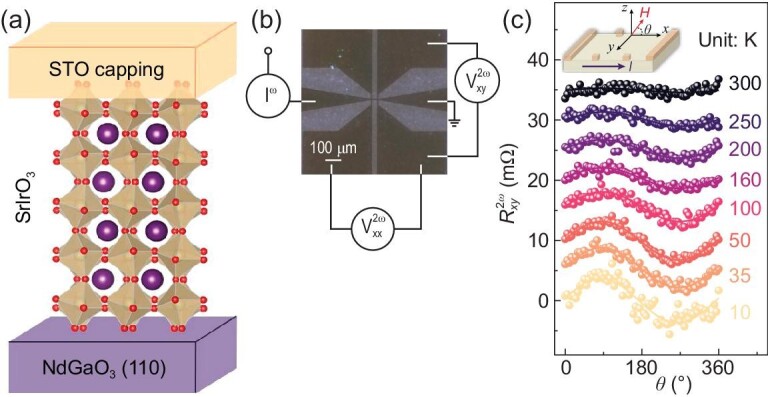
Observation of NLMR in a 24-u.c. SIO film. (a) A schematic of SIO/NGO (110) films. (b) Microscopic image of the fabricated Hall-bar device. (c) Magnetic-field angular dependence of $R_{xy}^{2{\mathrm{\omega }}}$ in a 24-u.c. SIO film along the *x–z* plane. Inset indicates the measuring geometry.

A series of SIO films with different thicknesses (from 24 to 200 u.c.) were epitaxially grown on NdGaO_3_ (110) [NGO (110)] substrates by using pulsed laser deposition (see ‘Materials and methods’). To avoid possible degradation of the samples in the atmosphere, amorphous SrTiO_3_ capping layers were deposited (Fig. 2a). The measured SIO Hall-bar device was fabricated by using standard photolithography and Ar ion milling, with channel length *L* = 50 μm and width *W* = 20 μm (Fig. 2b). The temperature-dependent resistance (*R*–*T*), magnetoresistance (MR) as a function of the magnetic field and carrier mobility of SIO films all exhibit a clear semi-metallic behavior [33–35] (Fig. S1 in the Supplementary information). The annular dark-field scanning transmission electron microscopy (ADF-STEM) image indicates the atomically sharp interface between the NGO substrate and the SIO (inset of Fig. S1).

The NLMR signal was first revealed in the 24-u.c. SIO film. Figure 2c shows the magnetic-field angular dependence of $R_{xy}^{2\omega }$ along the *x–z* plane with varied temperatures. The angle *θ* between the field and the current direction (along *x*) is defined in the inset of Fig. 2c. The alternating current (∼1 mA) is applied along the [100]*_pc_*-direction (*pc* denotes pseudo-cubic) and the magnetic field is ∼9 Tesla. From 10 to 300 K, $R_{xy}^{2\omega }$ displays an obvious cosine angular dependence with a period of 2π, indicating the field-dependent non-reciprocal transport character [15,18]. The peak positions are at *θ* = 7π/18 (70°, along *z*) and stay unchanged with the varied temperatures. These results indicate the existence of spin texture in the 24-u.c. SIO film and the spin orientation on the Fermi contour has the out-of-plane (OOP) component. The magnetic-field angular dependence of $R_{xy}^{2\omega }$ with a current applied along the [010]*_pc_*-direction (Fig. S9) also indicates the OOP warping behavior. First-principles calculations (Fig. S18) indicate that the interface/surface of the SIO slab breaks inversion symmetry, creating spin splitting of the band structure, i.e. the interface-induced spin texture of the SIO. The spin components in the *z*-directions are non-zero, which indicates that the spin texture of the SIO has the OOP warping. These theoretical results are consistent with our experimental observations (Fig. 2c and Fig. S9) and previous theoretical results [28].

### Details of mapping the spin texture

**Figure 3.**
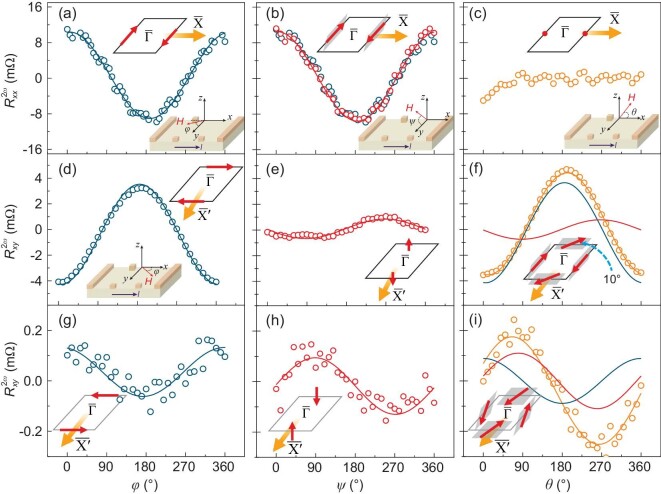
Mapping spin texture by NLMR of a 200-u.c. SIO film. (a)–(c) Magnetic-field angular dependence of second-order longitudinal resistance $R_{xx}^{2{\mathrm{\omega }}}$ at 10 K (*H* = 9 T, *I* = 1 mA). Solid curves are the fitting results by using the cosine function. The magnetic field was rotated within three typical planes, i.e. *x–y* plane [defined as *φ*, bottom inset of (a)], *y–z* plane [defined as *ψ*, bottom inset of (b)] and *x–z* plane [defined as *θ*, bottom inset of (c)]. The top inset in (a)–(c) illustrates the projection of the spin vector on the Fermi contour in the *x–y, y–z* and *x–z* planes, respectively. (d) and (e) Magnetic-field angular dependence of second-order transverse resistance $R_{xy}^{2{\mathrm{\omega }}}$ at 10 K (*H* = 9 T, *I* = 1 mA) using the three typical scans. Solid curves are the fitting results by using the cosine function. We note that the measuring geometry in (d) has a 90° offset [starting from *x* to *y*, bottom inset of (d)] with that in (a) (starting from *y* to –*x*). Inset of (f): spin texture on the Fermi contour at 10 K. (g) and (h) Angular dependence of $R_{xy}^{2{\mathrm{\omega }}}$ at 300 K in three different geometries. Solid curves are fitting results. Insets are sketches of the projection of the spin texture (g) in the *x–y* plane or (h) along the *z*-axis. (i) $R_{xy}^{2{\mathrm{\omega }}}$ as a function of *θ* at 300 K. The orange curve is the sum of the red and blue curves. The inset is a sketch of the spin texture at the Fermi contour at 300 K.

The interface-induced spin texture is expected to be unchanged with the variation in SIO thickness. Next, we determine the spin texture in the momentum space by the NLMR in a 200-u.c. SIO film. Figure 3a–c represents the magnetic-field angular dependence of the $R_{xx}^{2\omega }$ at 10 K while rotating the applied magnetic field **H** in the *x–y* (*φ*), *y–z* (*ψ*) and *x–z* (*θ*) planes, respectively. The externally applied alternating current **I** is 1 mA along the [100]*_pc_*-direction. For *x–y* and *y–z* scans, they all exhibit cosine angular dependence with a period of 2π and the magnitude of $R_{xx}^{2\omega }$ (i.e. $\Delta R_{xx}^{2\omega }$) is almost the same (Fig. 3b). As shown in Figs S2 and S3, $\Delta R_{xx}^{2\omega }$ scales linearly with both **I** and **H** whereas, for the *x–z* scan, the $R_{xx}^{2\omega }$ is nearly zero. These features unveil the spin orientations along $\overline {{{\bf \Gamma X}}} $ on the Fermi contour. $R_{xx}^{2\omega }$ reaches the maximum or minimum when the current is perpendicular to the magnetic field (*φ* = 0° or 180°, as schematically depicted in middle and right panels of Fig. 1c) and is zero when the direction of the current is aligned with the magnetic field (*φ* = 90° or 270°), indicating that the spin orientation in the *x–y* plane is along the *y*-direction (inset of Fig. 3a). $R_{xx}^{2\omega }$ approaches zero when the magnetic field is along the *z*-direction (*ψ* = 90° or 270°), suggesting the absence of an OOP component (inset of Fig. 3b). The nearly zero of $R_{xx}^{2\omega }$ when rotating *θ* suggests that the spin vector orients perpendicularly to the *x–z* plane. Therefore, Fig. 3a–c clarifies that the projection of the spin vector is along the in-plane (IP) direction and has a negligible component along the OOP direction. In other words, the spin orientation on the Fermi contour is always perpendicular to the $\overline {{{\bf \Gamma X}}} $ momentum-space line without any warping towards the OOP direction.

Meanwhile, the spin orientation along $\overline {{{\bf \Gamma X}}^{\prime}} $ could be revealed by measuring $R_{xy}^{2{\mathrm{\omega }}}$ when the direction of the current is still along $\overline {{{\bf \Gamma X}}} $. As shown in Fig. 3d–f, those three scans all exhibit non-reciprocal transport characters at 10 K. The peak position at *φ* = 180° or 0° (Fig. 3d) is consistent with the picture depicted in the middle and right panel of Fig. 1d, respectively. We then obtain the IP projection of the spin vector from the *x–y* scan on the Fermi contour (top inset of Fig. 3d). Apart from that, the scan along the *y–z* plane indicates the existence of the OOP projection of the spin vector because there is a peak when the magnetic field is along the *z*-direction (*ψ* = 90° or 270°) (see inset of Fig. 3e). Note that the *x–z* plane fitting curve (orange) corresponds to the sum of the *x–y* (blue) and *y–z* (red) plane fitting curves, indicating that the spin vector tilts out of the *x–y* plane with an angle of ∼10° (inset of Fig. 3f). Hence, the spin texture with the Fermi surface warping of the 200-u.c. SIO film could be well mapped by simultaneously measuring the magnetic-field angular dependence of the second-order longitudinal and transverse resistance, i.e. $R_{xx}^{2\omega }$ and $R_{xy}^{2\omega }$. The spin texture totally lies the IP along $\overline {{{\bf \Gamma X}}} $ and tilts towards the OOP with a very small angle along $\overline {{{\bf \Gamma X}}^{\prime}} $. This determined spin texture in the 200-u.c. SIO film is not consistent with the observed (Fig. 2c) and calculated one (Fig. S18) induced by the broken interface/surface symmetry, in which both the $\overline {{{\bf \Gamma X}}} $ and $\overline {{{\bf \Gamma }}{{{\bf X}}}^{{\bf ^{\prime}}}} $ directions have OOP warping.

Figure 3g–i shows the magnetic-field angular dependence of the transverse second-order resistance $R_{xy}^{2\omega }$ at 300 K. The magnetic field was rotated within these typical three planes, as shown in the inset of Fig. 3d, b and c, respectively. The magnetic field is 9 Tesla and the alternating current is 1 mA. Compared with the data at 10 K, the peak position of the $R_{xy}^{2\omega }$ at 300 K has a clear phase shift. In particular, the phase shift is ∼ π for the *x–y* and *y–z* scans, and ∼ π/2 for the *x–z* scan. Such behavior has not been discovered in 24-u.c. SIO (Fig. 2c) or any other systems [14,19,28,36]. We could directly exclude this observation, which is related to the existence of magnetic ordering in the 200-u.c. SIO film by conducting the normal Hall measurement (Fig. S1). Moreover, the Fermi surface reconstruction mechanism [37] could also be excluded since the *n*-type carrier does not change with varied temperatures (Fig. S1). The Nernst effect could be also excluded because of its isotropic nature, i.e. $\rho _{xy}^{2{\mathrm{\omega }}}$/$\rho _{xx}^{2{\mathrm{\omega }}}{\mathrm{\ }}$= 1. Together with the data of $R_{xx}^{2{\mathrm{\omega }}}$ at 300 K (Fig. S4), we determine the spin orientations on the Fermi contour in the **k**-space, as depicted in the inset of Fig. 3i. Along both the $\overline {{{\bf \Gamma X}}} $ and $\overline {{{\bf \Gamma X^{\prime}}}} $ directions, the spin orientations have the OOP components, i.e. the OOP warped spin texture, which is consistent with the interface-induced spin texture. Compared with the 24-u.c. SIO, this observed behavior suggests that the spin texture in the 200-u.c. SIO has an entirely different origin.

### Strain gradient in thick SIO films

**Figure 4.**
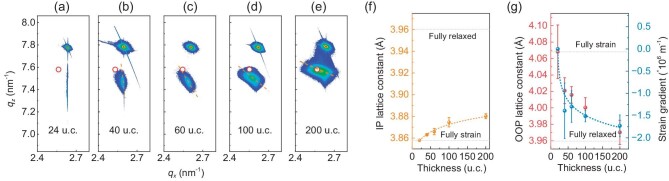
Strain gradient in SIO/NGO films. (a)–(e) Reciprocal space mapping (RSM) of SIO (103) films with varied thicknesses. The lattice parameter (pseudo-cubic) of the bulk value is labeled by red circles. Orange dashed lines indicate the tilt of the RSM patterns. (f) In-plane lattice parameter, extracted from RSM results, as a function of the SIO thickness. (g) Out-of-plane lattice parameter (red spheres) and computational strain-gradient value from the Williamson–Hall plot (blue spheres) as a function of the SIO thickness. Fully strained and fully relaxed values are labeled by gray dashed lines.

To investigate the origin of the tuned spin texture in the 200-u.c. SIO film, we systematically characterized the structure of the SIO films with varied thicknesses. As shown in Fig. 4a–e and Fig. S11, reciprocal space mapping (RSM) results show that the center of the SIO patterns moves continuously. The 24-u.c. SIO film is coherently strained to the NGO substrate, while the thick films show broadened peak centers. Figure 4f and g shows the IP and OOP lattice constants of the SIO films extracted from the RSM results. The IP lattice constant of the SIO films changes slowly while the OOP lattice constant changes quickly; the average OOP lattice constant of 200-u.c. SIO is nearly 3.97 ± 0.015 Å, which is close to the SIO bulk value (∼3.96 Å, indicated by red circles). X-ray diffraction and Raman measurements of SIO with varied thicknesses could also prove the change in the lattice constant, as shown in Fig. S13. Besides, the rotating angle of the peak center of the SIO film in RSM (green lines in Fig. 4a–e and Fig. S12) is highly correlated with the change in the metal–non-metal crossover temperature (*T*_cross_) in the *R–T* curves (Fig. S14). Those above analyses indicate the existence of a strain gradient in thick SIO films on NGO substrates [38,39] and the possible origin could be attributed to the mismatch of thermal coefficients (see Supplementary data). We could exclude the inhomogeneous strain gradient induced by defects since the STEM image (Fig. S1) indicates the high quality of the SIO film and sharp interface. Quantitative analysis of the strain gradient could be obtained by using a Williamson–Hall plot (Fig. S15); the results are shown in Fig. 4g (blue spheres). It has the same evolution trend as the OOP lattice constant and increases as the SIO thickness increases, until –1.75e^6^ m^−1^ for 200-u.c. SIO, which is one order of magnitude higher than La_1-x_Sr_x_MnO_3_ (LSMO) films grown on NGO [38]. Together with analysis [40] based on the triple-Gaussian fit of RSM (Fig. S17), it could be concluded that the 24-u.c. SIO film is fully strained, whereas the 200-u.c. SIO sample exhibits the most significant strain gradient. The strain gradient could be considered as the continuous change in the lattice constant across the different layers, resulting in a symmetry broken between each adjacent SIO layer and leading to a modified spin texture.

### Strain-gradient-induced spin texture

**Figure 5.**
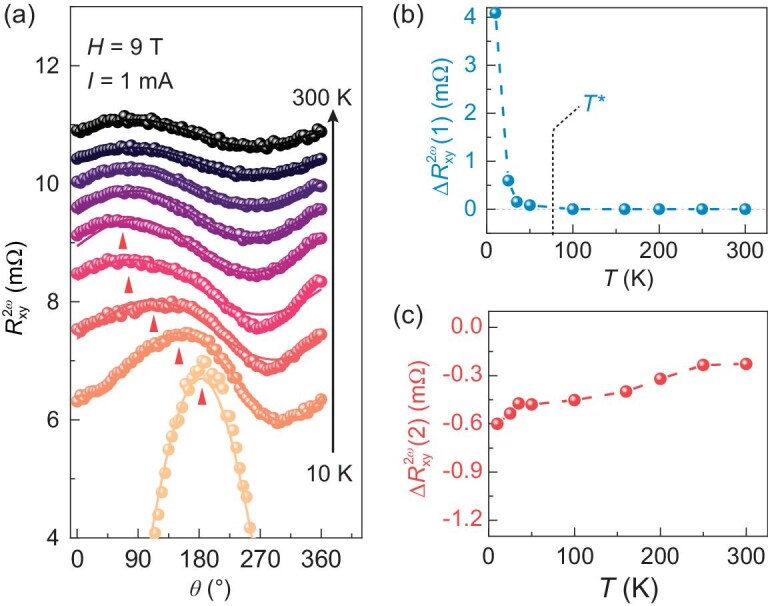
Two branches of second-order resistance. (a) Magnetic-field angular dependence of $R_{xy}^{2{\mathrm{\omega }}}$ measured at varied temperatures from 10 to 300 K (*H* = 9 T, *I* = 1 mA). Solid lines are fitting results by using the formula $R_{xy}^{2{\mathrm{\omega }}}$=${\mathrm{\Delta }}R_{xy}^{2{\mathrm{\omega }}}$(1)·cos(*θ*+19π/18) + ${\mathrm{\Delta }}R_{xy}^{2{\mathrm{\omega }}}$(2)·cos(*θ*+7π/18), where $R_{xy}^{2{\mathrm{\omega }}}$(1) and $R_{xy}^{2{\mathrm{\omega }}}$(2) are two branches of second-order transverse resistance. ${\mathrm{\Delta }}R_{xy}^{2{\mathrm{\omega }}}$(1) and ${\mathrm{\Delta }}R_{xy}^{2{\mathrm{\omega }}}$(2) represent their amplitudes, respectively. Peak shifts are indicated by red arrows. (b) and (c) ${\mathrm{\Delta }}R_{xy}^{2{\mathrm{\omega }}}$(1) and ${\mathrm{\Delta }}R_{xy}^{2{\mathrm{\omega }}}$(2), extracted from (a), as a function of temperature. Blue dashed lines are shown to guide the eyes. ${\mathrm{\Delta }}R_{xy}^{2{\mathrm{\omega }}}$(1) is prominent at lower temperatures and vanishes at a critical temperature T*, which is labeled in (b).

The discrepancy between the interface-induced spin texture (Fig. 2c and Fig. S18) and the mapped spin orientations on the Fermi contour at 10 K (inset of Fig. 3f), as well as the temperature-driven phase shift in the magnetic angular dependence of the second-order resistance in the 200-u.c. SIO film, could be attributed to the spin texture being tuned due to the strain gradient. To resolve the competition between the spin texture due to the inversion symmetry broken at the interface and that induced by the strain gradient, we analyse the detailed evolution of NLMR signals at varied temperatures. Figure 5a shows the $R_{xy}^{2{\mathrm{\omega }}}$ of the 200-u.c. SIO film as a function of the rotating angle at different temperatures, with the current applied along the [100]*_pc_*-direction. The phase shifts from 180° to 90° continuously from 10 to 100 K (as indicated by triangular arrows) and remains unchanged between 100 and 300 K. We try to use bi-cosine function to fit our results:


(1) \begin{eqnarray*}
R_{xy}^{2\omega } &=& \Delta R_{xy}^{2\omega }(1)\cos (\theta + 19\pi /18)\\ &&+ \Delta R_{xy}^{2\omega }(2)\cos (\theta + 7\pi /18)
\end{eqnarray*}

where ${\mathrm{\Delta }}R_{xy}^{2{\mathrm{\omega }}}$(1) and $R{\mathrm{\Delta }}_{xy}^{2{\mathrm{\omega }}}$(2) represent the amplitudes of the two second-order resistance signals (Fig. S7) and 19π/18 (190°) and 7π/18 (70°) correspond to the peak position at 10 and 300 K, respectively. The results are in good agreement with the experimental data and the changes in ${\mathrm{\Delta }}R_{xy}^{2{\mathrm{\omega }}}$(1) and ${\mathrm{\Delta }}R_{xy}^{2{\mathrm{\omega }}}$(2) as a function of temperature are shown in Fig. 5b and c. ${\mathrm{\Delta }}R_{xy}^{2{\mathrm{\omega }}}$(1) is prominent at lower temperatures and vanishes at a critical temperature T* between 75 and 100 K, which will be discussed later, while ${\mathrm{\Delta }}R_{xy}^{2{\mathrm{\omega }}}$(2) can exist until 300 K. Note that, at low temperatures, the value of ${\mathrm{\Delta }}R_{xy}^{2{\mathrm{\omega }}}$(1) is five times higher than that of ${\mathrm{\Delta }}R_{xy}^{2{\mathrm{\omega }}}$(2). Besides, ${\mathrm{\Delta }}R_{xy}^{2{\mathrm{\omega }}}$(1) and ${\mathrm{\Delta }}R_{xy}^{2{\mathrm{\omega }}}$(2) both scale linearly with **I** and **H**, as shown in Fig. S8. Extra data on $R_{xy}^{2{\mathrm{\omega }}}$ and $R_{xx}^{2{\mathrm{\omega }}}$ along other geometries (Figs S2–S6) and measured with the current applied along the [010]*_pc_*-direction (Fig. S10) are self-consistent and could lead to the same conclusion. Based on the above analyses, we conclude that two distinct branches of NLMR signals coexist in the thick SIO films, each originating from a different spin texture and competing with each other. The one that can remain at 300 K is caused by the inversion symmetry broken at the interface. The other, which dominates at low temperatures, is caused by the strain gradient.

**Figure 6.**
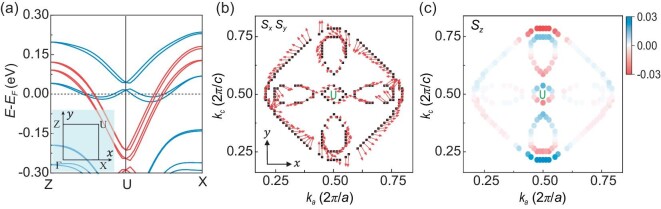
First-principles calculations for strain-gradient-induced spin texture. (a) The calculated band structures with the SOC of the SIO bulk model with the strain gradient. Inset presents Brillouin zone and high-symmetry k-point. (b) Spin textures on the *k_a_*–*k_c_* plane (*k_a_*||*x, k_c_*||*y*) at the Fermi surface for the SIO with the strain gradient. Local spin structures are shown by projecting the contributions from one Ir layer. The direction and length of the red arrow indicate the direction and magnitude of the spin vector, respectively. (c) The distribution of the spin component *S_z_*. Blue and red indicate the two directions of *S_z_*, and the shade of color indicates the magnitude of *S_z_*.

We perform the first-principles calculations to obtain the electronic band structure and the spin texture for the SIO bulk, which are shown in Fig. 6 and Fig. S19. The continuous change in the Ir atom distances in the ***b*** direction, i.e. the strain gradient, breaks the inversion symmetry and creates large spin splitting in the band structure, as shown in Fig. 6a. The spin texture on the *k_a_*–*k_c_* plane (*k_b_* = 0) is shown in Fig. 6b and c, which is different from the case of the fully strained state (Fig. S18). Near the *k_c_* = 0.5 momentum line ($\overline {{{\bf \Gamma X}}} $), the spin orientation is moving in the *x–y* plane, while the *S_z_* value is almost negligible, whereas, near the *k_a_* = 0.5 momentum line ($\overline {{{\bf \Gamma X^{\prime}}}} $), the spin vectors have a component along the *z*-direction. From the crystal structure, the SIO with the strain gradient has *M_y_* symmetry. Along the Z–U **k**-path, the *M_y_* symmetry makes the *S_z_* zero, while the non-zero *S_z_* is allowed along the X–U **k**-path. Therefore, the spin orientation on the Fermi contour only has an IP component along $\overline {{{\bf \Gamma X}}} $ and tilts the OOP along $\overline {{{\bf \Gamma X^{\prime}}}} $, which is consistent with the warping behaviors observed experimentally (inset of Fig. 3f).

### Discussion

We have shown that the spin texture in the momentum space of nominally centrosymmetric SIO could be caused by symmetry being broken at the interface and in the bulk of the film introduced by the strain gradient. These two effects lead the effective tuning of the spin texture, demonstrated by our magnetic-field angular dependence of NLMR measurements and theoretical calculations. We note that the calculated spin-splitting energy of the interface Rashba states (Fig. S18) is ∼200 *k*_B_, while the spin-splitting energy calculated from the band structures (Fig. 6a) is ∼82.5 *k*_B_, which is consistent with the observed T* and effectively describes the experimental phenomena dominated by the strain gradient at low temperatures. This description quantitatively agrees well with phenomena observed at both high and low temperatures. The strain gradient is a lattice effect, which is more likely influenced by the thermal fluctuation or disorder [41].

In previous studies, through chemical doping [15], electrical gating [17,19] or varying the substrates to change the strain states [28], the second-order resistance could not be modulated. This is because NLMR signals are solely determined by the spin texture in the momentum space. These methods cannot tune the spin texture in the momentum space, demonstrating its robust nature. However, we realize the effective tuning of the spin texture by introducing the strain gradient, which competes with the interface-induced one, thereby adjusting the NLMR. Moreover, previous theoretical works suggested that NLMR only occurs in systems with helical warped Fermi surfaces. However, our observations indicate that spin texture can also exist and be effectively tuned in a nominally 4-fold centrosymmetric material, which needs other interpretations, such as spin-momentum-locking inhomogeneities [42] and the generation of a pseudo magnetic field [17].

## CONCLUSION

In conclusion, we have investigated NLMR and demonstrated how to induce spin texture in **k**-space of nominally centrosymmetric materials. Our experiments showed that NLMR can be observed in a fully strained SIO film, indicating the existence of spin texture, which is a consequence of the strong SOC and inversion symmetry broken at the interface. In the case of the SIO films under the strain gradient, we used NLMR to map the spin texture in detail and found that the Fermi contour warping behavior is distinct from the interface-induced spin texture observed in the fully strained sample. Our band structure calculations indicated that the continuous change in the lattice constant across the different layers due to the strain gradient results in the symmetry being broken between each adjacent SIO layer, creating large spin-split bands. The competition between the interface-induced and the strain-gradient-induced spin splitting gives rise to two branches of NLMR signals. Our work demonstrates that the strain gradient can efficiently induce nontrivial spin textures in **k**-space and suggests that this mechanism may be applicable to any system with strong SOC and inversion symmetry.

## MATERIALS AND METHODS

SrIrO_3_ films were grown by using pulsed laser deposition on NdGaO_3_ (110) substrates (KrF excimer laser, λ = 248 nm). The growth was monitored by using *in situ* reflection high energy electron diffraction (RHEED) and the thickness was determined by counting the number of RHEED oscillations. For the second-order signal measurements, an AC current *I*_ω_ = *I·*sin(*ωt*) was applied by using a Keithley 6221 current source while measuring the transverse AC harmonic Hall voltage and extracting the second harmonic resistance *R*^2ω^ from *V*^2ω^ by using a lock-in amplifier (SR830, Stanford Research) at a frequency of 17.7 Hz. The details of the experiment are given in the online Supplementary material.

## Supplementary Material

nwad296_Supplemental_File
