# Supplementary material for: Momentum-space spin texture induced by strain gradient in nominally centrosymmetric SrIrO3 films
Source: Natl Sci Rev. 2023 Nov 21;11(10):nwad296. doi: 10.1093/nsr/nwad296 (PMC11409885; doi:10.1093/nsr/nwad296)
Supplement: nwad296_Supplemental_File [file nwad296_supplemental_file.pdf]

Supplementary Data:

Momentum-space spin texture induced by strain gradient in nominally centrosymmetric $SrIrO_3$ films

Minghui Gu[1,2†], Haohao Sheng[1,2†], Xiaofeng Wu[1,2], Mei Wu[3], Xiaoran Liu[1], Fang Yang[1], Zhongshan Zhang[1], Peng Gao[3], Zhijun Wang[1,2*], Meng Meng[1*], Jiandong Guo[1,2*]

[1] *Beijing National Laboratory for Condensed Matter Physics and Institute of Physics, Chinese Academy of Sciences, Beijing, China.*

[2] *School of Physical Sciences, University of Chinese Academy of Sciences, Beijing, China.*

[3] *International Center for Quantum Materials, and Electron Microscopy Laboratory, School of Physics, Peking University, Beijing, China.*

[†]These authors contributed equally to this work.
*To whom correspondence should be addressed:
wzj@iphy.ac.cn; mengm@iphy.ac.cn; jdguo@iphy.ac.cn;

This PDF file includes:

Supplementary Text
Figs. S1 to S19
Tables S1
References (1 to 22)

Supplementary Text

Materials and Methods

Sample preparation. A series of $SrIrO_3$ with different thickness were grown by pulsed laser deposition (PLD) on $NdGaO_3$ (110) substrates (KrF excimer laser, $\lambda = 248$ nm). The base pressure was better than $2e^{-6}$ Pa. The growth was monitored by *in-situ* reflection high energy electron diffraction (RHEED) and the thickness was determined by counting the number of RHEED oscillations. The laser fluence and repetition rate were set as 2 J/cm^2 and 1 Hz, respectively. The oxygen partial pressure was optimized at 10 Pa and the growth temperature was 670 °C, respectively. To avoid possible degradation of the samples in atmosphere, amorphous STO capping layers were deposited at room temperature after growth and annealing 1 h at 10 Pa oxygen atmosphere.

RSM, XRD and Raman measurements. Reciprocal space mapping (RSM) and X-ray diffraction (XRD) measurements were performed by using a Rigaku SmartLab (9 kW) X-ray diffractometer with a Ge (400) ×2 crystal monochromator. Raman spectra were collected by using a confocal Raman microscope (Horiba HR Evolution) with a 325 nm laser as the excitation source. The polarizer and analyzer are under parallel polarization geometry.

Device fabrication and Transport measurements. We use standard UV lithography to form a micro-fabricated Hall bar pattern with channel length 20 μm. The electrical transport properties were revealed by a Physical Property Measurement System (PPMS,

Quantum Design). For the second-order signal measurements, an AC current $I_{\omega}=I\cdot\sin(\omega t)$ were applied by using Keithley 6221 current source while measuring the transverse AC harmonic Hall voltage and extracting the second harmonic resistance $R^{2\omega}$ from $V^{2\omega}$ by using lock-in amplifier (SR830, Stanford Research) at a frequency of 17.7 Hz.

STEM measurements. For scanning transmission electron microscopy (STEM) image acquisition, the cross-sectional STEM specimens were thinned to less than ~30 μm first by using mechanical polishing and then by performing argon ion milling. The ion-beam milling was carried out using PIPSTM (Model 691, Gatan Inc.) with the accelerating voltage of 3.5 kV until a hole was made. Low voltage milling was performed with accelerating voltage of 0.3 kV to remove the surface amorphous layer and to minimize damage. High-resolution high-angle annular dark-field (HAADF) images were recorded at 300 kV using an aberration-corrected FEI Titan Themis G2 with the convergence semi-angle for imaging 30 mrad and the collection semi-angles snap 39 to 200 mrad.

First-principles calculations. We carried out the first-principles calculations within the framework of the density functional theory (DFT) using projector augmented wave (PAW) method[1, 2], as implemented in the Vienna *ab initio* simulation package (VASP)[3, 4]. The generalized gradient approximation (GGA) of Perdew-Burke-Ernzerhof (PBE) type[5] was employed for the exchange-correlation potential. The kinetic energy cutoff for plane wave expansion was set to 500 eV. For $SrIrO_3$ slab, the thickness of the vacuum layers along *b* directions was set to > 20 Å. The Brillouin zone

was sampled by Γ-centered Monkhorst-Pack method in the self-consistent process, with a 8×8×8 *k*-mesh for $SrIrO_3$ bulk and a 8×1×8 *k*-mesh for $SrIrO_3$ slab. The spin-orbit coupling (SOC) was taken into account in all calculations.

Principles of second-order signal measurements

When a first-order alternating current (I^{ω}, $\propto J_{e}^{\omega}$) passes through materials with strong SOC, according to Taylor expansion, R_{xy} (using transverse resistance as an illustration) could be written as a function of I^{ω}:

$$R_{xy} = R_{xy}^{(1)} + R_{xy}^{(2)} * I^{\omega} + o(I^{\omega}),$$

where $R_{xy}^{(1)}$ is the ordinary Hall resistance, which is independent to I^{ω}. $R_{xy}^{(2)}$ is the second-order resistance, and $o(I^{\omega})$ is higher order components and could be omitted. In addition, the voltage could be written as:

$$V_{xy} = \left(R_{xy}^{(1)} + R_{xy}^{(2)} * I^{\omega}\right) * I^{\omega} = R_{xy}^{(1)} * I^{\omega} + R_{xy}^{(2)} * (I^{\omega})^2 = R_{xy}^{(1)} * I\sin\omega t + R_{xy}^{(2)} * I^2\sin^2\omega t \ = R_{xy}^{(1)} * I\sin\omega t + \frac{1}{2}I^2R_{xy}^{(2)} + \frac{1}{2}I^2R_{xy}^{(2)}\sin\left(2\omega t - \frac{\pi}{2}\right),$$

in which $\frac{1}{2}I^2R_{xy}^{(2)}$ is a dc component, $R_{xy}^{(1)} * I\sin\omega t$ is the first-order term, and $\frac{1}{2}I^2R_{xy}^{(2)}\sin\left(2\omega t - \frac{\pi}{2}\right)$ is the second-order signal, respectively. Usually, $R_{xy}^{(1)}$ is one or two order of magnitudes larger than $R_{xy}^{(2)}$, which needs lock-in amplifiers to extract the signals. The second-order resistance is defined as $R_{xy}^{2\omega}=\frac{1}{2}I^2R_{xy}^{(2)}/I \ =\frac{1}{2}IR_{xy}^{(2)}$.

Extra electrical transport data of 200-u.c. SIO

Temperature dependent resistivity (*ρ*-*T*) curve shows clear semimetallic behavior with the resistivity around 1 mΩ·cm [**Fig. S1(a)**], which has the same order of magnitude as previous reports[6]. The magnetoresistance (MR) measured between ±2 T at 10 K is positive and has the

same range of variation as literature reports[7, 8], which is shown in **Fig. S1(b)**. Hall measurements [**Fig. S1(c)**] shows the n-type carrier character and the absence of hysteresis loop, implying the non-magnetic behavior of SIO. Besides, the charge carrier density and mobility can be extracted and shown in **Fig. S1(d)**, which has the same order of magnitude with previous reports[7, 8], further reflecting the semimetallic characters of our SIO sample.

NLMR results with current applied along [010]-direction

To investigate the role of crystal anisotropy on the **k**-space spin textures, we have grown another 24-u.c. SIO film on NGO substrate and fabricated into Hall bar device, as shown in **Fig. S9**. In this sample, current could be applied along both [100] and [010] direction, simultaneously. **Fig. S9 (b) and (c)** shows the magnetic-field angular dependences of $R_{xy}^{2\omega}$ rotated in x-z plane with varied temperatures when current is applied along [100] and [010] direction, respectively. With current along both directions, all curves display an obvious cosine angular dependence with a period of 2π, indicating that the existence of out-of-plane spin texture along both directions, which is consistent well with the feature of spin texture of the 24 u.c. SIO film shown in **Fig. 2 (c)** and the 200 u.c. SIO film at 300 K shown in **Fig. 3**.

We have grown another 200-u.c. SIO film on NGO substrates and fabricated into the Hall bar device, as shown in **Fig. S10**. In this sample, current could be applied along both [100] and [010] direction, simultaneously. Firstly, we set alternating current along [100] directions and measure the magnetic-field angular dependences of $R_{xy}^{2\omega}$ along x - z plane, as shown in **Fig. S10 (b)**. The distinct peak shifts (as shown in **Fig.5** in the main text) demonstrate the reproducibility. Secondly, we set alternating current along [010] direction and measure the magnetic-field angular dependences of $R_{xy}^{2\omega}$ along three geometries, as shown in **Fig. S10 (c)**

- (e). The NLMR signals change sign between 10 K and 50 K, consistent with that data presented in Fig. S2&3 measured with current along [100]. Besides, it has the same magnitude of $R_{xy}^{2\omega}$ when rotating φ and ψ, together with the result of nearly zero $R_{xy}^{2\omega}$ when rotating θ, suggesting the spin vector orients perpendicularly to the *x*-*z* plane without any warping towards to the OOP direction. These results draw the same conclusion as shown in Fig. 3 (a) - (c) with the current applied along [100], and clarify that for NLMR measurements in SIO, measuring $R_{xy}^{2\omega}$ with current along [010] has the same results as measuring $R_{xx}^{2\omega}$ with current along [100]. Thirdly, we also measure the magnetic-field angular dependences of $R_{xx}^{2\omega}$ along three geometries at 10 K, as shown in Fig.S10 (f) - (h) and draw the same conclusion as shown in Fig. 3 (d) - (f). This is because when an electric field $\boldsymbol{E}$ is applied, nonlinear spin current is generated simultaneously at both the longitudinal and transverse directions due to the perpendicularly spin-momentum locking. In other words, the NLMR signals are only relevant to the measurement direction of second-harmonic voltage but irrelevant to current direction. These results also reveal the spin texture is robust and plays a decisive role on NLMR signals.

Details of structure characterizations and strain gradient

Strain gradient is a fancy type of stress-strain relationship in epitaxial films with fully-strained near substrates and partially-relaxed or fully-relaxed far from substrates. This mechanism usually attracts attentions on flexoelectricity area[9-13] in transition metal oxides, such as $HoMnO_3$[14]. Recently, scientists found that apart from flexoelectricity, strain gradient also affects other physical properties, such as tuning magnetic easy axis[15] and stabilizing room temperature skyrmions[16].

Strain gradient in SIO thin films with varied thickness was evaluated by XRD spectra , as

shown in **Fig. S15(a)**. The out-of-plane strain gradient can be estimated by $E_{zz,z} = -\varepsilon_i/\delta$ based on Williamson-Hall plot, as shown in **Fig. S15(b)**. Here, δ is the out-of-plane penetration depth of the strain in SIO/NGO films, which is estimated to be ~100 nm, close to the value of LSMO[16]. ε_i is the out-of-plane inhomogeneous strain of each SIO/NGO film determined from the slope of the Williamson-Hall plot as:

$$\beta \cos(\theta) = \frac{K\lambda_w}{D} + 4\varepsilon_i \sin(\theta)$$

where θ is Bragg angle, β=β_{measured} - $\beta_{\text{instrument}}$, with β_{measured} being the measured breadth of the diffraction peaks of the thin films and $\beta_{\text{instrument}}$ estimated from the breadth of the nearby substrate reflection. $K\lambda_w/D$ is a constant. The fitting curves and strain gradients are exhibited in **Fig. S15(b)** and **Fig. S15(c)** respectively, where the slopes of curves give ε_i for thin films with different thickness.

Strain gradient analyzed by RSM has been utilized in different systems [17-19]. Except for the Williamson-Hall plot method, which can be used to obtain the estimation value of strain gradient quantitively [16], using the method described in Ref. 17, we made further analysis based on RSM data. As shown in **Fig. S17(a)**, the *c*/*a* ratio of SIO film is changed gradually with increasing thickness, indicating a gradual reduction of strain. Besides, the asymmetric (103) reflections of 40 - 200 u.c. SIO samples enclose a narrow streak at q_x values of NGO substrate [**Fig. 4 (a) - (e)** in the main text], indicating the presence of a pseudomorphic layer of SIO at the interface with NGO. This layer is highly stressed, and the crystal lattice parameter *a* is identical to the one of NGO. In turn, this leads to a strongly elongated *c*-axis. In addition, a non-elliptical shape of the reflection suggests the presence of structural inhomogeneity along out-of-place direction, i.e., a strain gradient.

We use the 40-u.c. SIO as an illustration because of the distinct pseudomorphic region. We split the RSM into sections parallel to q_x [A, B and C, **Fig. S17 (b)**] and q_z axes [D, E and F, **Fig. S17 (b)**] and fit their profiles separately using triple-Gaussian. We analyze the variation of the intensities and positions of individual peaks to assess the lattice parameters of SIO in three structurally different regions along out-of-plane direction: (i) close to the bottom interface, (ii) in the interior of the film and (iii) close to the top surface. All sections could be well-fitted by triple-Gaussian, indicating it has three different regions with different lattice constants, i.e., pseudomorphic region (blue), transition region (red) and top region (green), respectively [**Fig. S17 (e)&(h)**]. This is the key character of the strain gradient. Therefore, RSM data is capable to give robust evidence of the existing strain gradient in SIO/NGO films.

Discussions about the origin of strain gradient

The lattice constant of STO substrate is 3.905 Å and lattice structure of STO is cubic. For LSAT, it has same lattice structure with STO but the lattice constant is 3.868 Å. NGO (3.858 Å, orthorhombic) has almost similar lattice constant with LSAT but different lattice structure, so we can investigate possible origin of strain gradient by comparing samples grown on STO, NGO and LSAT. No relaxation was found in SIO/STO samples because no peak shift was observed in both XRD and RSM spectrum (**Fig. S16(a)-(d)**). While for SIO/LSAT samples, it is fully relaxed (**Fig. S16(e)-(h)**). In other words, strain gradient does not exist in SIO/STO and SIO/LSAT. We believe lattice constant of substrate as well as lattice symmetry are both indistinguishable in formation of strain gradient.

SIO and NGO are both orthorhombic structure, which means they have different thermal coefficient along ***a***, ***b***, ***c*** axis, as shown in **Table 1**. But for LSAT, thermal coefficients are same

and smaller than that of NGO, so the higher thermal coefficient mismatch of SIO and LSAT may eliminate the possibility of strain gradient and cause fully relax.

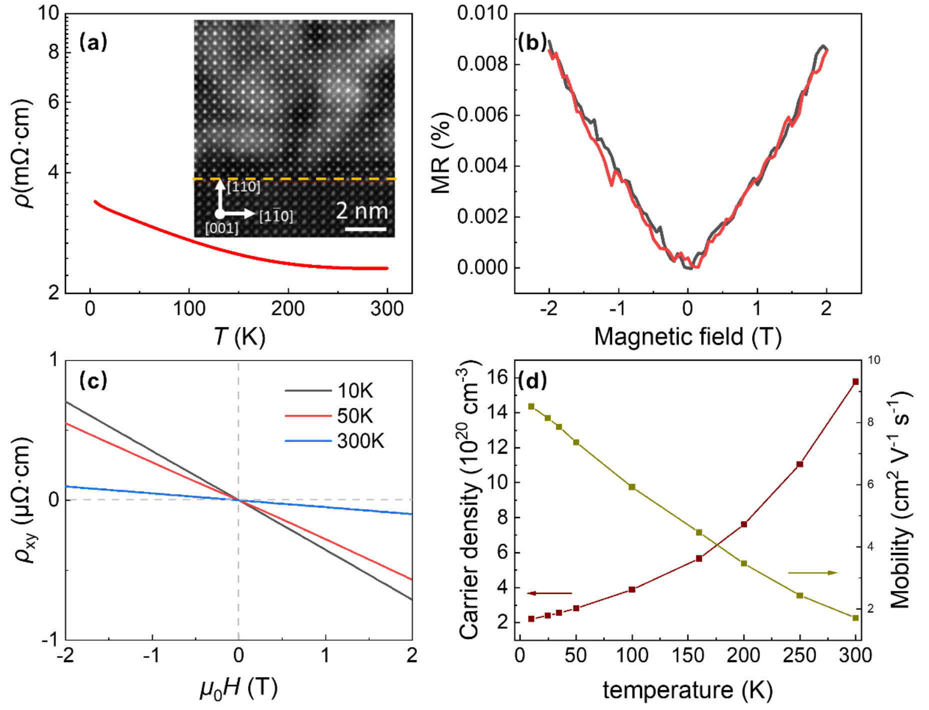

Fig. S1. Electrical transport of a 200-u.c. SIO film. **a** temperature-dependent resistance (*R*-*T*) curve. Inset is the STEM image of 200-u.c. film, indicating sharp interface. **b** magnetoresistance (MR) curve measured at 10 K between ±2 T. Red curve: +2 T to -2 T. Black curve: -2 T to +2 T. **c** transverse electrical transport (Hall) curves under different temperatures. **d** carrier density and mobility extracted from **c**.

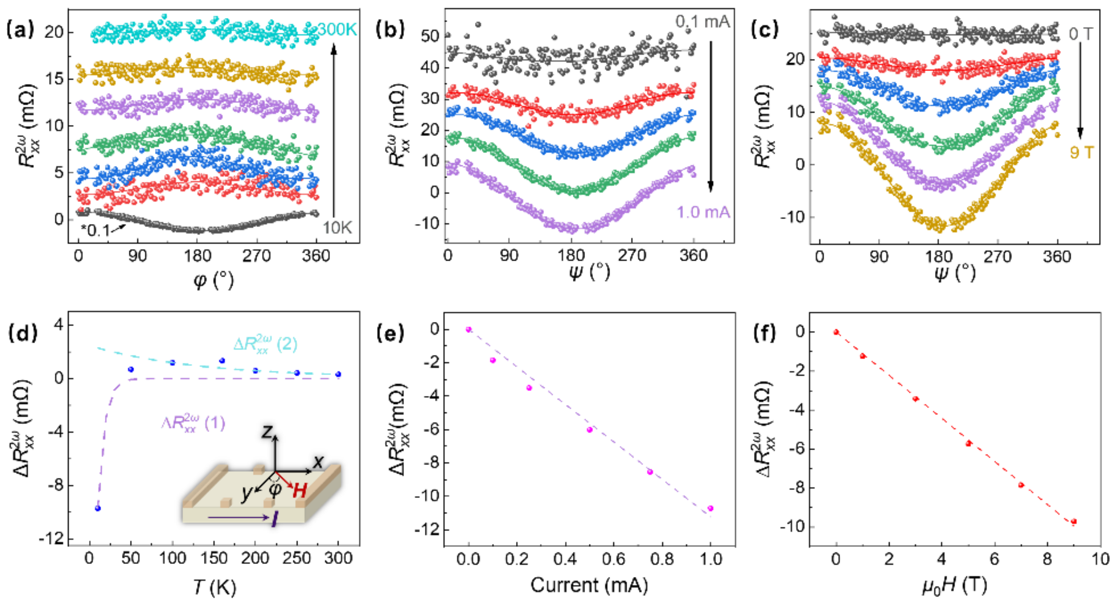

Fig. S2. Longitudinal second-order resistance $R_{xx}^{2\omega}$ as a function of rotating *H* along *x-y* plane (defined as φ). **a-c** $R_{xx}^{2\omega}$ with varied temperatures (10 K - 300 K at 9 T and 1 mA, **a** $R_{xx}^{2\omega}$ at 10 K is divided by 10), varied currents (0.1 mA - 1.0 mA at 10 K and 9 T, **b**) and varied magnetic fields (0 T - 9 T at 10 K and 1 mA, **c**). Solid lines are fitting results by using cosine function. **d** $\Delta R_{xx}^{2\omega}$ extracted from **a**. $\Delta R_{xx}^{2\omega}(1)$ and $\Delta R_{xx}^{2\omega}(2)$ are two series of second-order resistance. Purple and cyan dash lines are shown to guide eyes. **e-f** $\Delta R_{xx}^{2\omega}$ extracted from **b** or **c**, dashed line is linear fitting result.

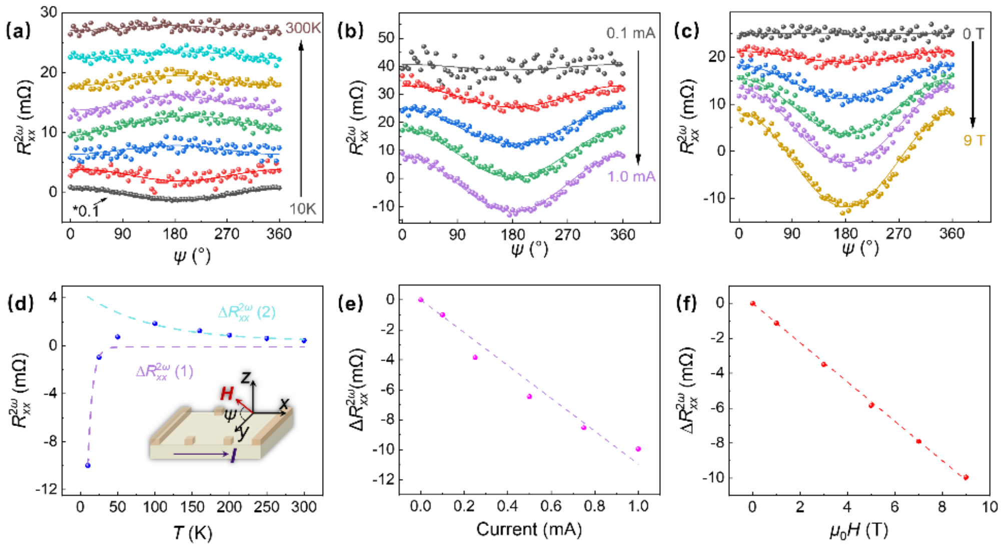

Fig. S3. Longitudinal second-order resistance $R_{xx}^{2\omega}$ measured with rotating *H* along *y*-*z* plane (defined as *ψ*). **a-c** $R_{xx}^{2\omega}$ with varied temperatures (10 K - 300 K at 9 T and 1 mA, **a** $R_{xx}^{2\omega}$ at 10 K is divided by 10), varied currents (0.1 mA - 1.0 mA at 10 K and 9 T, **b**) and varied magnetic fields (0 T - 9 T at 10 K and 1 mA, **c**). Solid lines are fitting results by using cosine function. **d** $\Delta R_{xx}^{2\omega}$ extracted from **a**. $\Delta R_{xx}^{2\omega}(1)$ and $\Delta R_{xx}^{2\omega}(2)$ are two series of second-order signals. Purple and cyan dash lines are shown to guide eyes. **e-f** $\Delta R_{xx}^{2\omega}$ extracted from **b** or **c**, dashed line is linear fitting result.

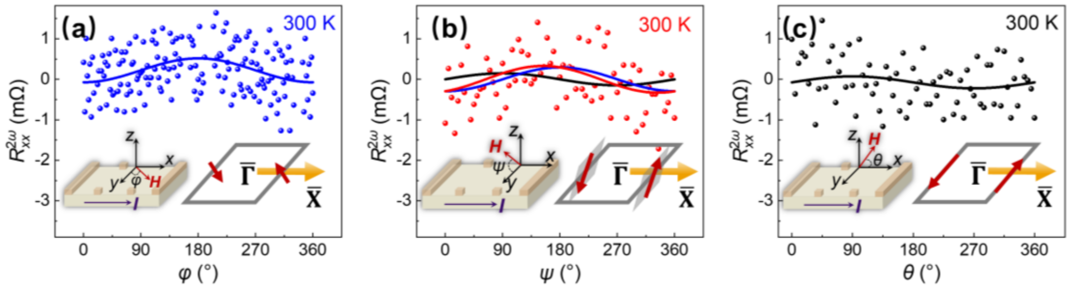

Fig. S4. Longitudinal second-order resistance $R_{xx}^{2\omega}$ measured with rotating *H* along three scans at 300 K and 9 T along $\overline{\Gamma X}$ direction. Inset are the rotation geometries and Fermi contour warping illustrations.

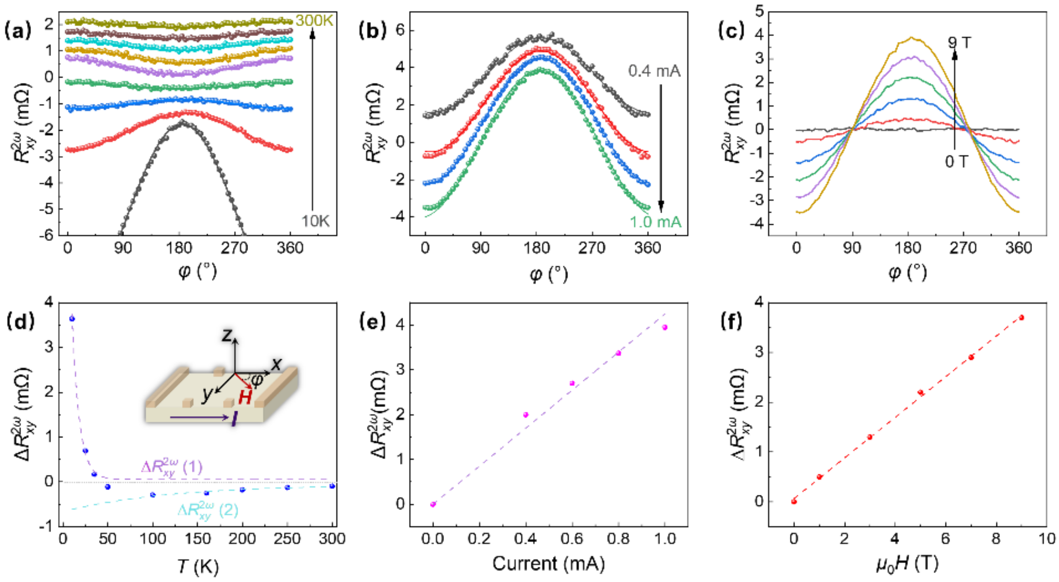

Fig. S5. Transverse second-order resistance $R_{xy}^{2\omega}$ with rotating H along x-y plane (defined as φ). **a-c** $R_{xy}^{2\omega}$ with varied temperatures (10 K - 300 K at 9 T and 1 mA, **a**), varied currents (0.1 mA - 1.0 mA at 10 K and 9 T, **b**) and varied magnetic fields (0 T - 9 T at 10 K and 1 mA, **c**). Solid lines are fitting results by using cosine function. **d** $\Delta R_{xy}^{2\omega}$ extracted from **a**. $\Delta R_{xy}^{2\omega}(1)$ and $\Delta R_{xy}^{2\omega}(2)$ are two series of second-order signals. Purple and cyan dash lines are shown to guide eyes. **e-f**, $\Delta R_{xy}^{2\omega}$ extracted from **b** or **c**, dashed line is linear fitting result.

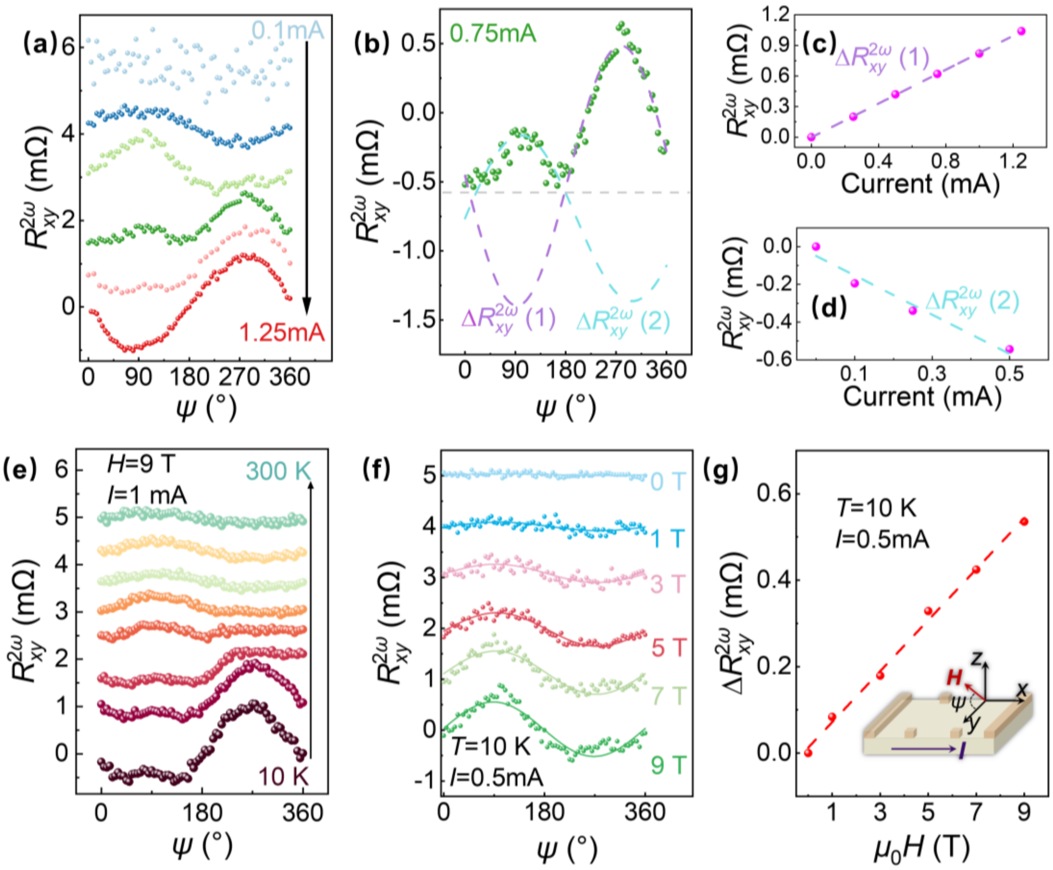

Fig. S6. Transverse second-order resistance $R_{xy}^{2\omega}$ measured with rotating *H* along *y-z* plane (defined as *ψ*). **a** $R_{xy}^{2\omega}$ with varied currents (0.1 mA - 1.25 mA at 9 T and 10 K). **b** the fitting results of two series of second harmonic signals using 0.75 mA as an illustration. **c-d** $\Delta R_{xy}^{2\omega}(1)$ and $\Delta R_{xy}^{2\omega}(2)$ as a function of current. Dashed lines are linear fitting results. **e** $R_{xy}^{2\omega}$ with varied temperatures (10 K - 300 K at 9 T and 1 mA). **f** $R_{xy}^{2\omega}$ with varied magnetic fields (0 T - 9 T at 10 K and 0.5 mA). Solid lines are fitting results by using cosine function. **g** $\Delta R_{xy}^{2\omega}$ extracted from **f**. Dashed line is linear fitting result.

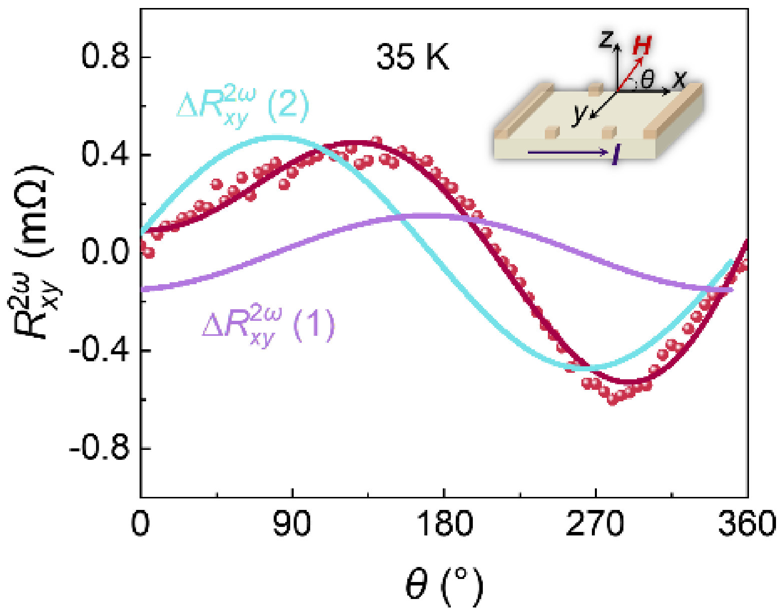

Fig. S7. The method to fit the second-order signals along *x*-*z* scan, using data at 35 K as an illustration. Red spheres show the raw data. Red curve is total fitting curve by using bi-cosine fitting function $R_{xy}^{2\omega}$=$\Delta R_{xy}^{2\omega}$(1)·cos(θ+π) + $\Delta R_{xy}^{2\omega}$(2)·cos(θ+π/2). Purple and cyan curves show two series of second-order signals, respectively. Inset is the illustration of scanning geometry.

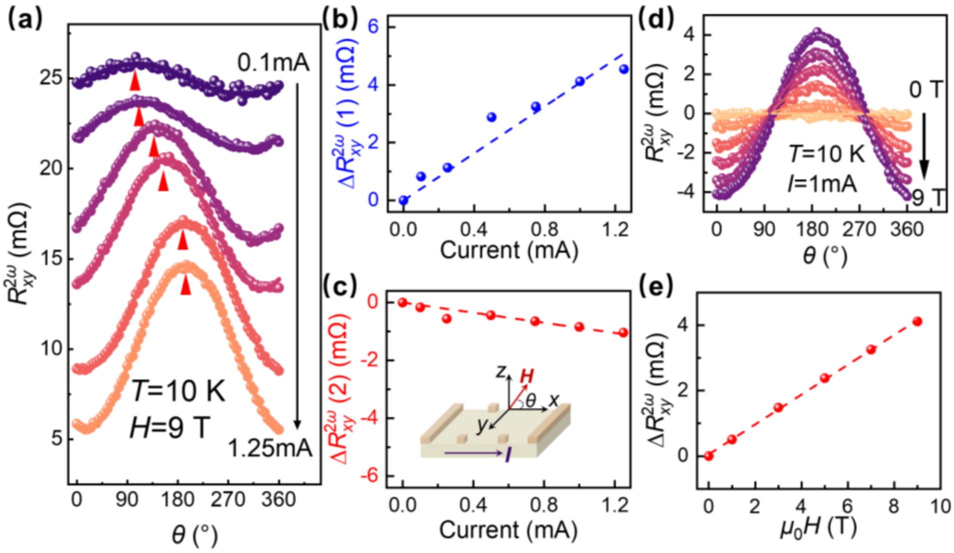

Fig. S8. **a**, angular dependence of $R_{xy}^{2\omega}$ measured at 0.1 - 1.25mA (*H* = 9 T, *T* = 10 K) with rotating *H* along *x*-*z* plane (defined as *θ*), solid lines are fitting results. Phase shifts are marked by red triangle arrows. **b-c** $R_{xy}^{2\omega}(1)$ and $R_{xy}^{2\omega}(2)$ (extracted from **a**) as a function of current. **d** angular dependence of $R_{xy}^{2\omega}$ at 0 - 9 T (*T* = 10 K, *I* = 1 mA), solid lines are fitting results. **e** $R_{xy}^{2\omega}$(extracted from **d**) as a function of magnetic field.

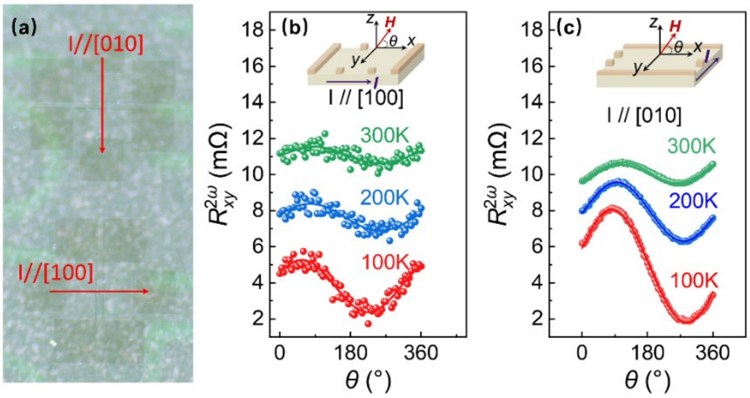

Fig.S9. a Hall bar device of a 24-u.c. SIO film obtained by standard lithography and etching process. Different current directions are marked. The magnetic-field angular dependences of $R_{xy}^{2\omega}$ along *x*-*z* plane with varied temperatures when current is set along **b** [100] direction and **c** [010] direction.

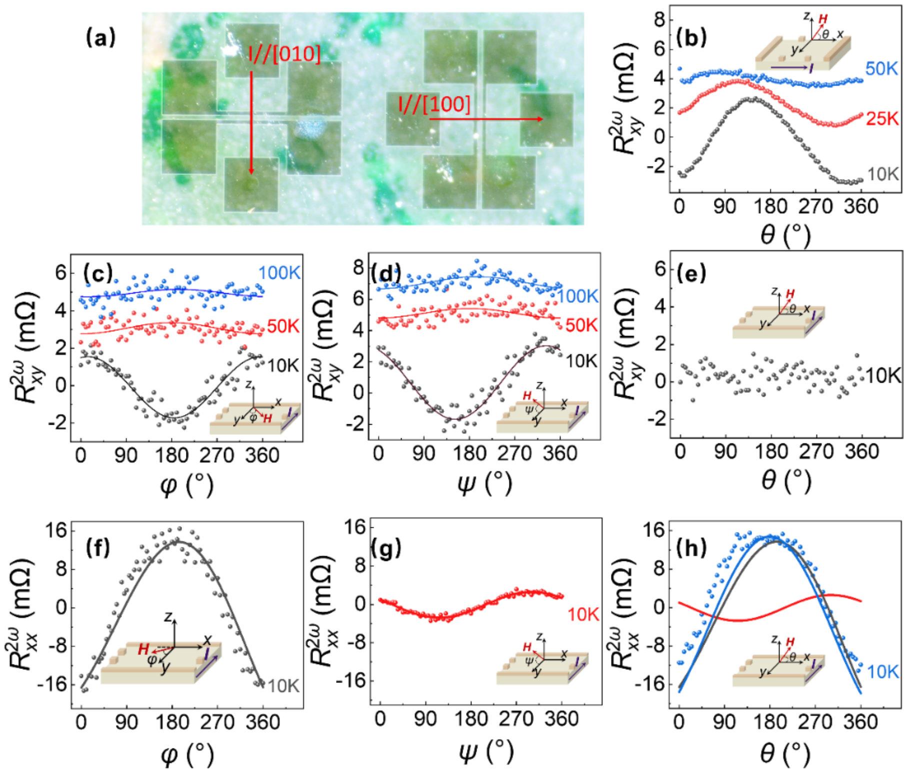

Fig.S10. a Hall bar device of a 200-u.c. SIO film obtained by standard lithography and etching process. Different current directions are marked. **b** The magnetic-field angular dependences of $R_{xy}^{2\omega}$ along *x-z* plane with varied temperatures when current is along [100] direction (*H* = 9 T, *I* = 1 mA). **c - e** The magnetic-field angular dependences of $R_{xy}^{2\omega}$ along **c** *x-y* plane, **d** *y-z* plane, and **e** *x-z* plane with varied temperatures when current is along [010] direction. **f - h** The magnetic-field angular dependences of $R_{xx}^{2\omega}$ along **f** *x-y* plane, **g** *y-z* plane, and **h** *x-z* plane with varied temperatures when current is along [010] direction.

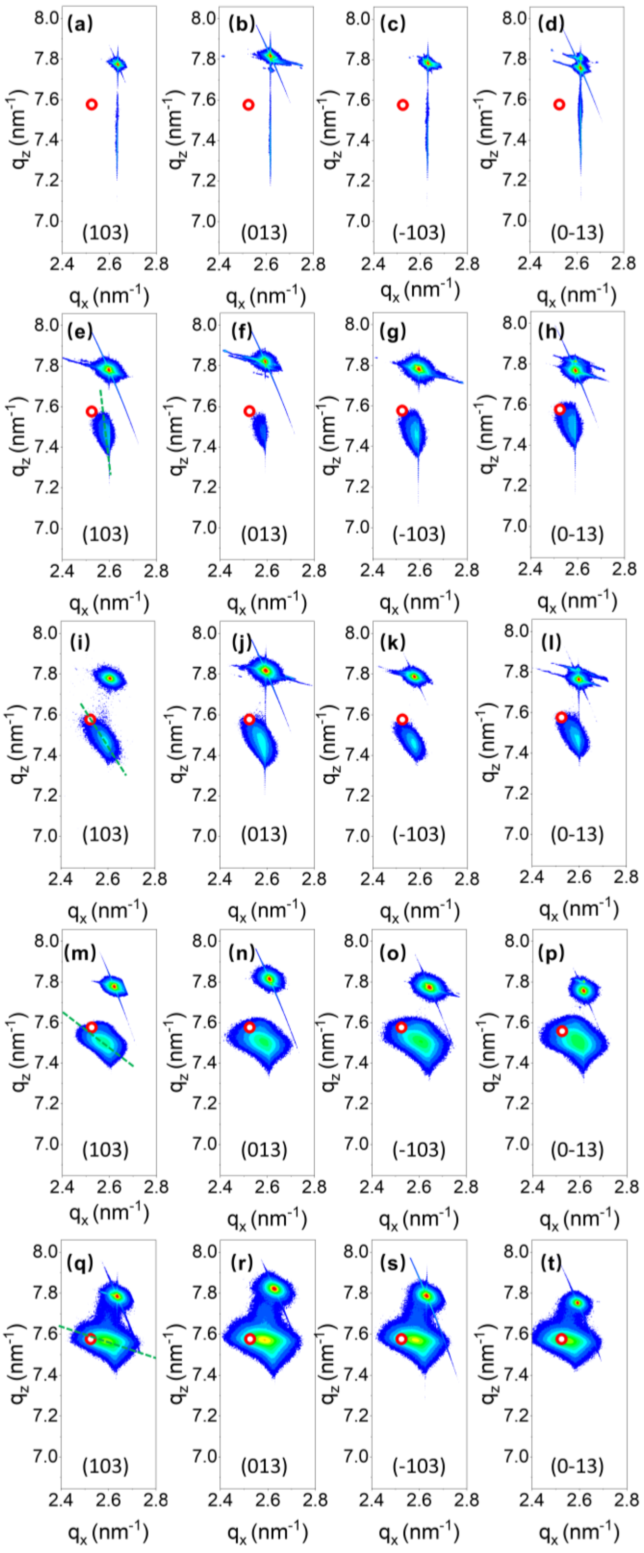

Fig. S11. RSM results with four planes in **a - d** 24-u.c., **e - h** 40-u.c., **i - l** 60-u.c., **m - p** 100-u.c. and **q - t** 200-u.c. SIO. Red circles are positions of SIO bulk values.

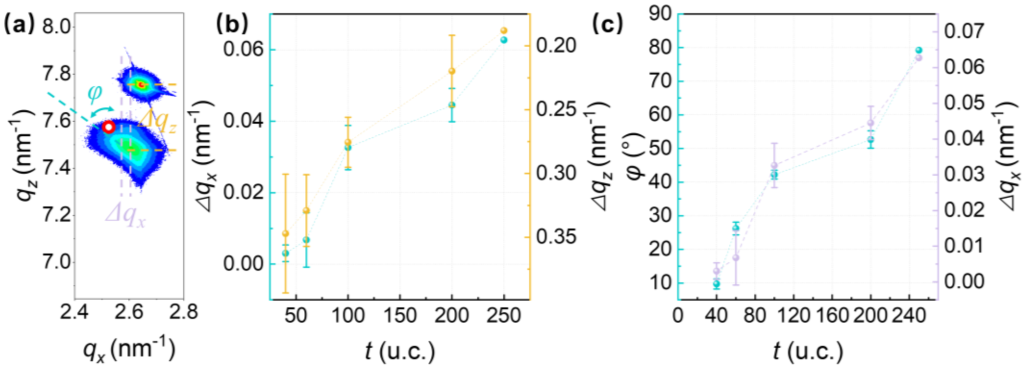

Fig. S12. Statistical details of Δq_x, Δq_z and φ. **a** The definition of Δq_x, Δq_z and φ. Δq_x (Δq_z) is defined as the deviation between q_x (q_z) of SIO film and NGO substrate. φ is defined as the rotating angle of the RSM center of SIO comparative to vertical direction. **b** The change of Δq_x (Δq_z) as a function of film thickness (t). **c**, The change of φ and Δq_x as a function of t, they all show the same evolution trend.

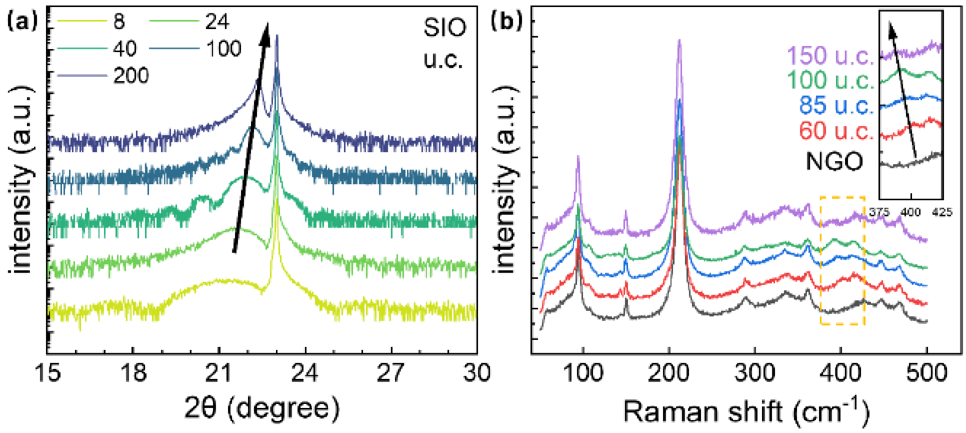

Fig. S13. a XRD and **b** Raman results. Both show clear SIO peak shifts.

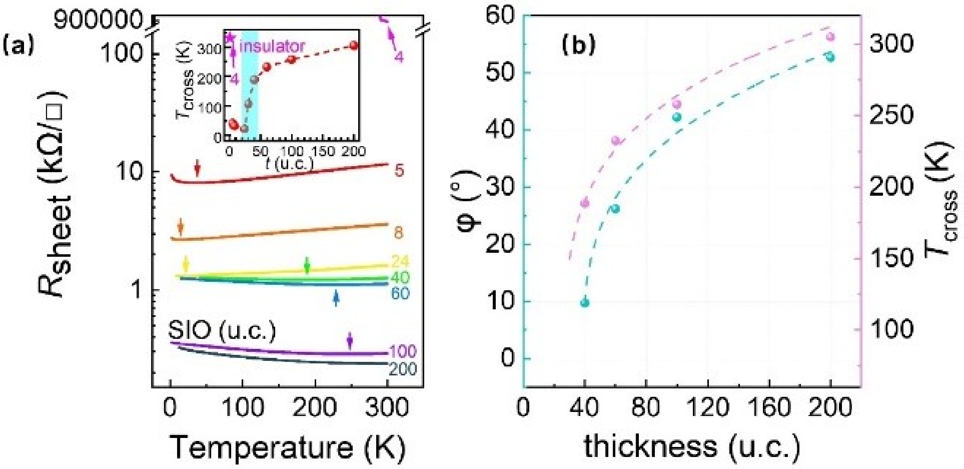

Fig. S14. The relationship between strain gradient and T_{cross}.

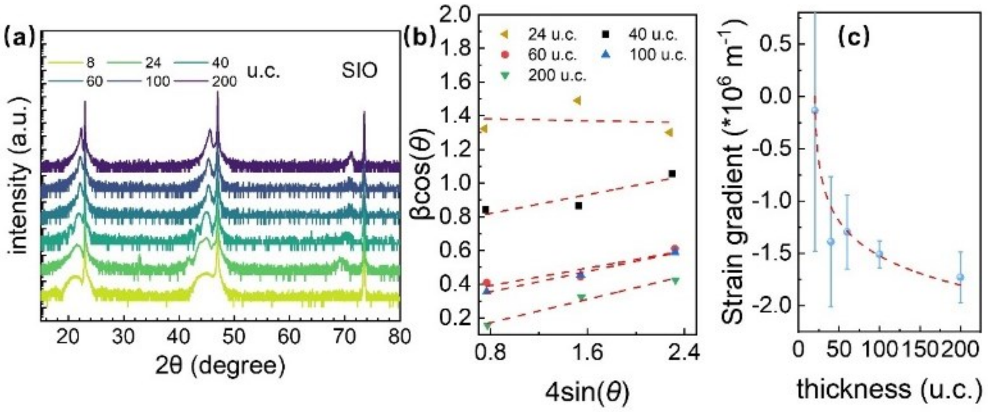

Fig. S15. Strain gradient fitting details by using Williamson-Hall plot.

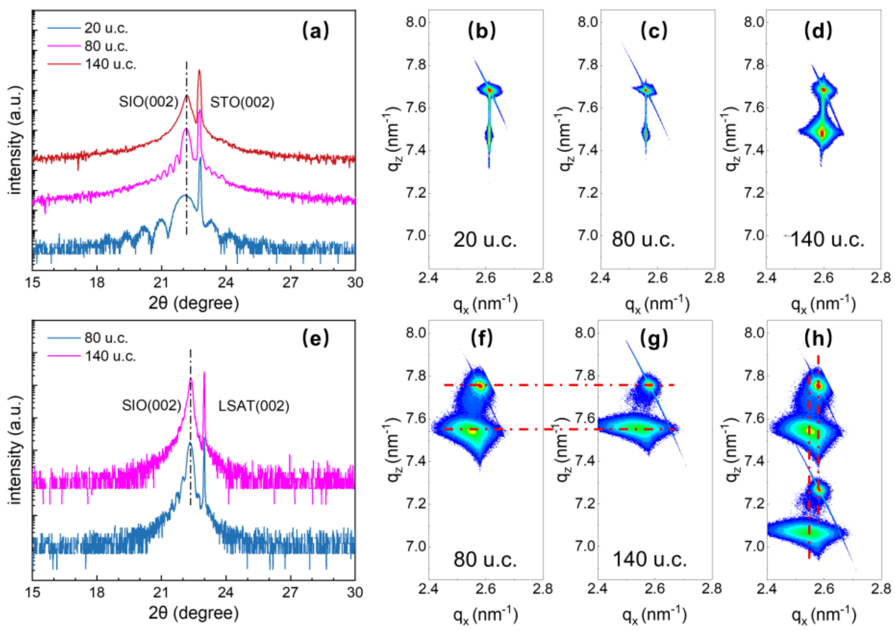

Fig. S16. XRD and RSM measurements of SIO grown on STO and LSAT substrates. **a** XRD (002) peaks of SIO grown on STO substrate with different thickness. **b - d** RSM measurements along STO (103) direction. **e** XRD (002) peaks of SIO grown on LSAT substrate with different thickness, indicating fully strain lattice of SIO. **f - g** RSM measurements along LSAT (103) direction. The comparison of Δq_x, Δq_z are shown by red dashed lines. Indicating fully relax lattice of SIO.

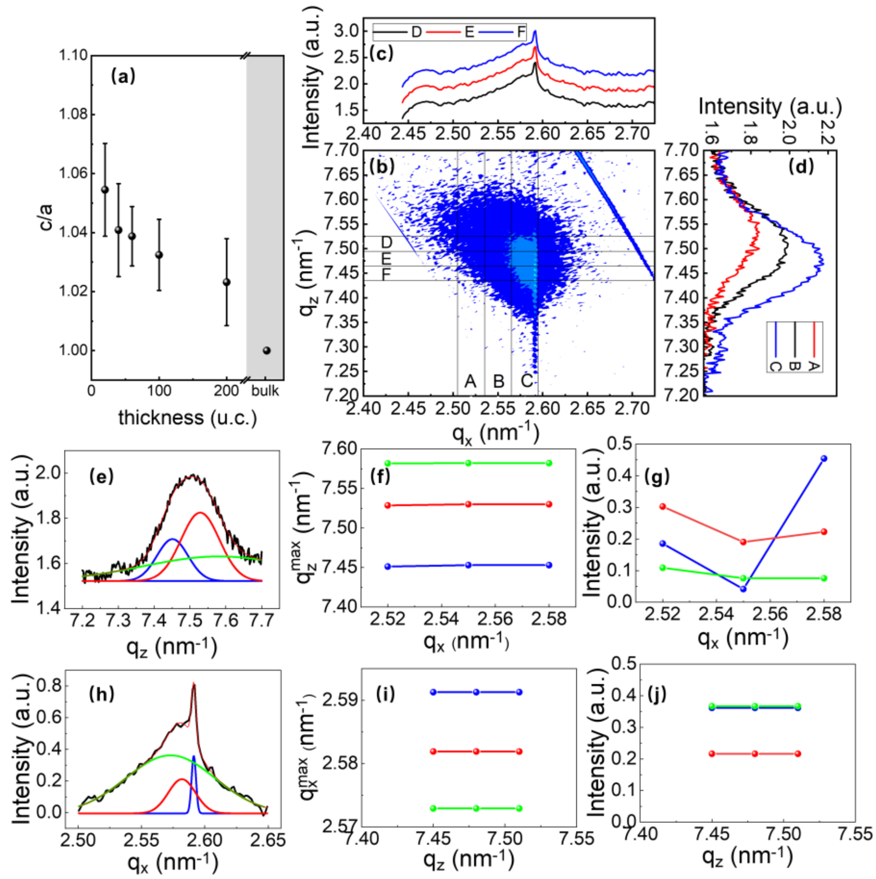

Fig.S17. a The *c/a* ratio of all SIO samples. *c/a* ratio of bulk strain-free SIO (~ 1) is taken as a reference. **b** The slicing RSM data along q_x and q_z axes along the black lines. The interslice separation is 0.03 nm along q_x and q_z axes. Each slice is indicated with a capital letter. **c&d** The set of line scans showing the change of the intensity of the reflection within the slice area along q_x and q_z axes. **e&h** The line scans of the averaged intensity along q_x and q_z axes. (black curves). The best fit to the experimental data can be done with triple Gaussian peaks, which are indicated with red, green and blue curves. **f&i** Peak positions extracted from **e** and **h**, showing the same for all slices. **g&j** Peak intensities extracted from **e** and **h**.

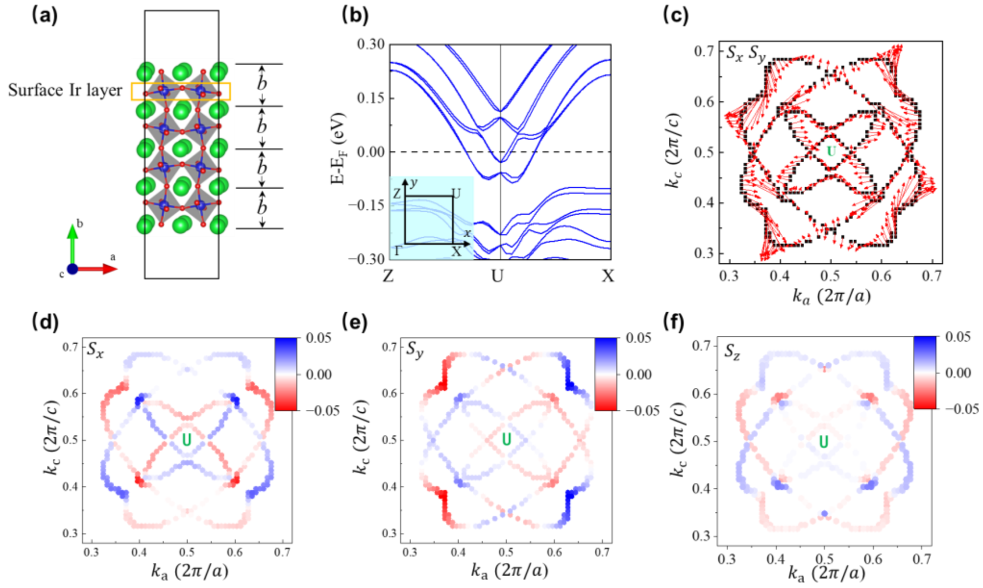

Fig. S18. a Crystal structure of the SIO slab. The green, blue, and red balls represent Sr, Ir, and O atoms, respectively. The gray oxygen octahedron is represented. **b** The band structures with SOC of the SIO slab. Inset presents Brillouin zone and high-symmetry **k**-point. **c** Theorical calculation of spin texture (S_x and S_y) on the k_a - k_c plane (k_a // x, k_c // y) at Fermi surface for the SIO slab. The direction and length of red arrow indicates the direction and magnitude of the spin vector, respectively. **d-f** Spin textures on the k_a-k_c plane at Fermi surface for the SIO slab, including the spin component S_x (**d**), S_y (**e**), and S_z (**f**). Local spin structures are shown by projecting the contributions from the surface Ir layer, which is marked in **a**. Blue and red indicate two directions of the spin, and the shade of color indicates the magnitude of the spin vector.

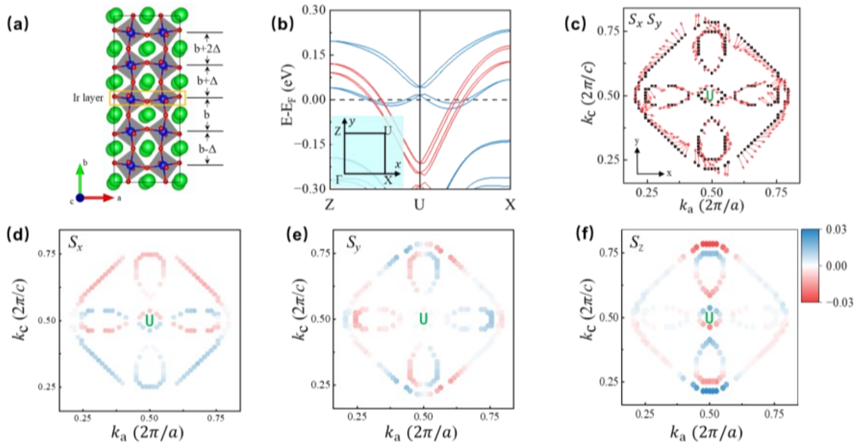

Fig. S19. a Crystal structure of the SIO bulk with strain gradient. *Δ* indicates the continuous change in lattice constant across different layers. The green, blue, and red balls represent Sr, Ir, and O atoms, respectively. The gray oxygen octahedron is represented. **b** The band structures with SOC of the SIO bulk. Inset presents Brillouin zone and high-symmetry **k**-point. **c** Theorical calculation of spin texture (S_x and S_y) on the k_a - k_c plane (k_a // x, k_c // y) at Fermi surface for the SIO slab. The direction and length of red arrow indicates the direction and magnitude of the spin vector, respectively. **d-f** The distribution of the spin component S_x, S_y, and S_z. Blue and red indicate two different directions, and the shade of color indicates the magnitude.

substrate	a axis	b axis	c axis
LSAT		$5.8*10^{-6}$/K	
NGO	$11.9*10^{-6}$/K	$6.6*10^{-6}$/K	$5.8*10^{-6}$/K
SIO	$1*10^{-6}$/K	$8*10^{-6}$/K (300K)<br>to<br>$22*10^{-6}$/K (1273K)	$2*10^{-6}$/K (300K)<br>to<br>$14*10^{-6}$/K (1273K)

Table S1. Thermal coefficient of LSAT, NGO and SIO along different axis[20-22].

Reference:

1. Blochl PE. Projector augmented-wave method. *Phys Rev B* 1994; **50**: 17953-17979.

2. Kresse G, Joubert D. From ultrasoft pseudopotentials to the projector augmented-wave method. *Phys Rev B* 1999; **59**: 1758-1775.

3. Kresse G, Furthmuller J. Efficiency of *ab-initio* total energy calculations for metals and semiconductors using a plane-wave basis set. *Comput Mater Sci* 1996; **6**: 15-50.

4. Kresse G, Furthmuller J. Efficient iterative schemes for *ab initio* total-energy calculations using a plane-wave basis set. *Phys Rev B* 1996; **54**: 11169-11186.

5. Perdew JP, Burke K, Ernzerhof M. Generalized gradient approximation made simple. *Phys Rev Lett* 1996; **77**: 3865-3868.

6. Groenendijk DJ, Autieri C, Girovsky J *et al.* Spin-Orbit Semimetal $SrIrO_3$ in the Two-Dimensional Limit. *Phys Rev Lett* 2017; **119**: 256403.

7. Nishio K, Hwang HY, Hikita Y. Thermodynamic guiding principles in selective synthesis of strontium iridate Ruddlesden-Popper epitaxial films. *APL Mater* 2016; **4**: 036102.

8. Lee D, Roh S, Hwang J *et al.* Engineering electrical property of Dirac semimetal perovskite $SrIrO_3$ thin films by subtle changes in lattice structure. *Appl Phys Express* 2020; **13**: 015510.

9. Zubko P, Catalan G, Tagantsev AK. Flexoelectric Effect in Solids. *Annu Rev Mater Res* 2013; **43**: 387-421.

10. Nguyen TD, Mao S, Yeh YW *et al.* Nanoscale flexoelectricity. *Adv Mater* 2013; **25**: 946-974.

11. Jeon BC, Lee D, Lee MH *et al.* Flexoelectric effect in the reversal of self-polarization and associated changes in the electronic functional properties of $BiFeO_3$ thin films. *Adv Mater* 2013; **25**: 5643-5649.

12. Lee D, Noh TW. Giant flexoelectric effect through interfacial strain relaxation. *Phil. Trans* 2012; **370**: 4944-4957.

13. Lee D, Yoon A, Jang SY *et al.* Giant Flexoelectric Effect in Ferroelectric Epitaxial Thin Films. *Phys Rev Lett* 2011; **107**: 057602.

14. Lee D, Yang SM, Yoon JG *et al.* Flexoelectric rectification of charge transport in strain-graded dielectrics. *Nano Lett* 2012; **12**: 6436-6440.

15. Peng W, Park SY, Roh CJ *et al.* Flexoelectric control of a ferromagnetic metal. *arXiv*. 2203.03199.

16. Zhang Y, Liu J, Dong Y *et al.* Strain-Driven Dzyaloshinskii-Moriya Interaction for Room-Temperature Magnetic Skyrmions. *Phys Rev Lett* 2021; **127**: 117204.

17. Makushko P, Kosub T, Pylypovskyi OV *et al.* Flexomagnetism and vertically

graded Neel temperature of antiferromagnetic Cr_2O_3 thin films. *Nat Commun* 2022; **13**: 6745.

18. Yamahara H, Feng B, Seki M *et al.* Flexoelectric nanodomains in rare-earth iron garnet thin films under strain gradient. *Commun Mater* 2021; **2**: 95.

19. Yang Y, Mao X, Yao Y *et al.* Thickness effects on the epitaxial strain states and phase transformations in (001)-VO_2/TiO_2 thin films. *J Appl Phys* 2019; **125**: 082508.

20. Sasaura M, Miyazawa S, Mukaida M. Thermal expansion coefficients of high-Tc superconductor substrate $NdGaO_3$ single crystal. *J Appl Phys* 1990; **68**: 3643-3644.

21. Longo JM, Kafalas JA, Arnott RJ. Structure and properties of the high and low pressure forms of $SrIrO_3$. *J Solid State Chem* 1971; **3**: 174-179.

22. Ohta J, Fujioka H, Sumiya M *et al.* Epitaxial growth of AlN on (La,Sr)(Al,Ta)O_3 substrate by laser MBE. *J Cryst Growth* 2001; **225**: 73-78.
